# A rare case of nevus sebaceous of the bilateral labia minora: A case report and review of literature

**DOI:** 10.1097/MD.0000000000025047

**Published:** 2021-03-12

**Authors:** Qiao Wen, Zhiwei Zhao, Yanlin Yang, Fengnian Zhao, Ling Wang, Juan Cheng, Jiang Wu, Yali Miao

**Affiliations:** a Department of Obstetrics and Gynecology, Key Laboratory of Birth Defects and Related Diseases of Women and Children of MOE, West China Second University Hospital; b Deep Underground Space Medical Center, West China Hospital, No. 37 Guoxuexiang; c West China School of Basic Medical Sciences & Forensic Medicine; d West China School of Clinical Medicine, Sichuan University, Chengdu, China.

**Keywords:** case report, genital area, labia minora, nevus sebaceous, prognosis, surgical resection

## Abstract

**Rationale:**

Nevus sebaceous (NS) is a lesion caused by congenital hyperplastic disorder of the sebaceous glands. It commonly noted in the scalp and face and rarely in the trunk, neck, or oral mucosa. We present a rare case of a lesion arising in the genital region.

**Patient concerns:**

A 47-year-old woman complained of a gradual increase in the size of her bilateral labia minora over 2 years, which affected her sexual life and caused walking difficulty. She was admitted to the Department of Obstetrics and Gynecology. On physical examination, no ulcer, discharge, and vulval or vaginal bleeding were found. The bilateral inguinal lymph nodes were not palpable, bilateral labia minora were asymmetric, and the right side was evidently bigger than the left. The labia minora had serrated edges and numerous papillae with a maximum diameter of 0.5 cm. The vagina, cervix, and uterus with its attachments were normal. Blood samples tested negative for human immunodeficiency virus, human papilloma virus, hepatitis B virus, and hepatitis C virus.

**Diagnosis:**

A diagnosis of NS of the bilateral labia minora was made following histopathological examination of the resected specimen.

**Intervention:**

The bilateral labia minora lesions were resected general anesthesia on August 29, 2016. The operation was successful, and intraoperative blood loss was about 10 ml.

**Outcomes:**

After 40 months of postoperative follow-up, no recurrence or appearance of other tumors were noted.

**Lessons:**

We recommend surgical removal of lesions in the genital area during adolescence or before adulthood. Adolescence may be the best period for surgical intervention owing to a greater risk of malignant change in adulthood. On the other hand, surgical risk should be avoided in children considering the low incidence of malignant transformation.

## 1 Introduction

Nevus sebaceous (NS) is often referred to as an organoid nevus, because it may contain any or all components of the skin, such as dermis, epidermis, and epidermal appendages. It is also called a sebaceous gland hamartoma and was first reported by Jadassohn in 1895. The onset of NS typically occurs at birth, and occasionally, in adulthood. In childhood, NS presents as an elevated hairless patch, that is pale yellow with a smooth surface and waxy luster. In adolescent patients, because the sebaceous gland can be fully developed by then, the nevus presents as nodular, petaloid or verrucous lesions. In elderly patients, NS presents as a brown, hard verrucous lesion.

NS most frequently occurs on the head and neck, and usually involves the scalp (87%–95% of cases) resulting in alopecia.^[1]^ It rarely occurs in other locations such as the eyelid and the oral mucosa, where it presents as plaques or nodules; multiple lesions are also a possibility.^[2–5]^ NS occurring in the genital organs is extremely rare, with only 5 cases reported in the literature to-date, out of which only 3 have been reported in detail.^[6,7]^ Since the specific morphological characteristics of genital organ NS are scarcely reported in the literature, clinical diagnosis is difficult, and cases can be easily misdiagnosed. This paper, with relevant literature review, elucidates the morphologic characteristics of labia minora lesions, that can aid plastic surgeons, dermatologists, and gynecologists to arrive at a suitable differential diagnosis and treatment strategy.

## 2 Case description

### 2.1 Chief complaints

A 47-year-old woman of Han ethnicity with obstetric history G2P0L1A1, who complained of gradually enlarging labia minora over 2 years, was admitted to the hospital on August 26, 2016.

### 2.2 History of present illness

Two years prior to admission, she found the right labia minora to have gradually enlarged; it was soft to touch and deep in color. There were no symptoms of swelling, erosion, or pain in the right labia minora. Subsequently, she found that the left labia minora was gradually enlarging too. The bilateral labia minora were asymmetrical, with the right side being larger than the left. Three months before admission, the labia minora enlarged rapidly, accompanied by mild tenderness that affected sexual life and walking to a certain extent. There were, however, no complaints of ulcer, discharge, and vulval or vaginal bleeding.

### 2.3 History of past illness

The patient had a 2-year history of hypertension and regularly took telmisartan 40 mg/d to maintain a blood pressure of 120 to 130/80 mm Hg.

### 2.4 Personal and family history

The patient maintained a normal diet, had no significant change in weight recently, and had no history of bowel or bladder dysfunction. Her menstrual cycles were normal; there was no history of radiation or poison exposure, previous surgeries, or relevant illnesses in the family.

### 2.5 Physical examination

**Figure 1:**
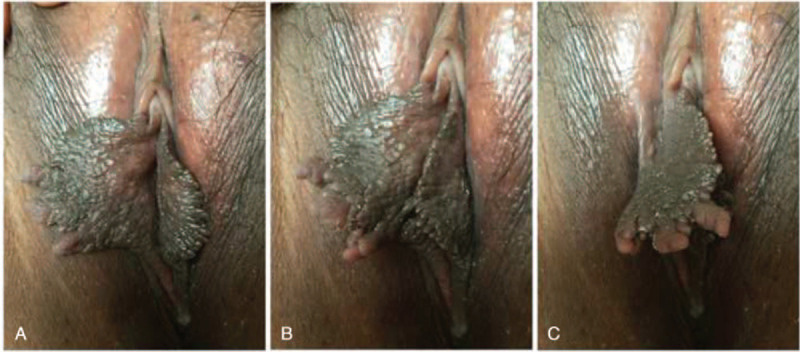
(A) Bilateral labia minora are asymmetric. (B) The right labia minora is evidently larger than the left. (C) The labia minora with a serrated edge presents multiple protrusions.

Systemic examination was found to be normal. On local examination, the bilateral inguinal lymph nodes were not palpable. The bilateral labia minora were asymmetric, with the right side being evidently bigger than the left. The labia minora had serrated edges and numerous papillae with a maximum diameter of 0.5 cm (Fig. 1A–C). The vagina, cervix, and uterus with its bilateral attachments were normal.

### 2.6 Laboratory examinations

Blood test results showed that the blood type was O (+). Moreover, tests for human immunodeficiency virus, human papilloma virus, syphilis, hepatitis B virus, and hepatitis C virus were all negative; serum cancer antigen 125 level was 13.35 U/ml and carbohydrate antigen 19-9 level was 7.41 U/ml. The cervical pap smear showed normal results.

### 2.7 Imaging examinations

A transvaginal color Doppler ultrasound was suggestive of a uterine adenomyoma. The results of electrocardiogram, chest X ray, and abdominal ultrasonography for liver and spleen were normal.

### 2.8 Final diagnosis

Before surgery, the dermatological consultant suggested removing the lesions, but did not provide a clear diagnosis. The postoperative pathological diagnosis was NS.

### 2.9 Treatment

The resection of bilateral labia minora lesions was performed under general anesthesia on August 29, 2016. The operation was successfully completed, and intraoperative blood loss was about 10 ml.

### 2.10 Outcome and follow-up

**Figure 2:**
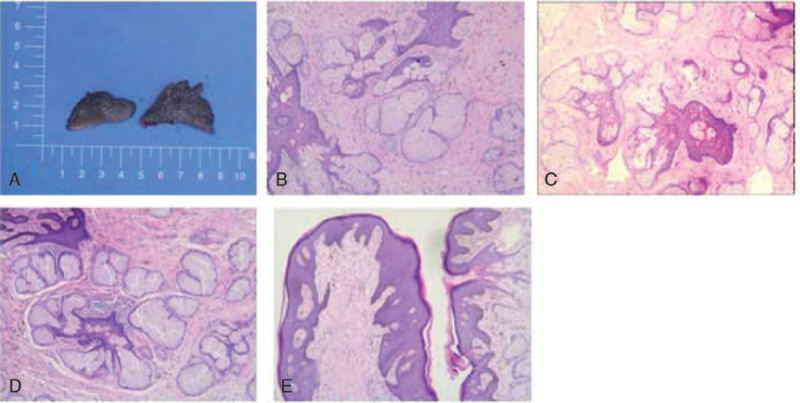
(A) Pathologic gross specimens of bilateral labia minora after surgical resection. The right lesion was 2.5 × 1.0 cm in size, and the left was 2.5 × 0.8 cm. (B–E) Plenty of enlarged sebaceous glands, slight hyperkeratosis, and acanthotic epidermis with papillomatosis. (B–D) microscopic findings of hematoxylin and eosin (H&E) staining, 40 × ; (E) microscopic findings of H&E staining, 10 × .

**Figure 3:**
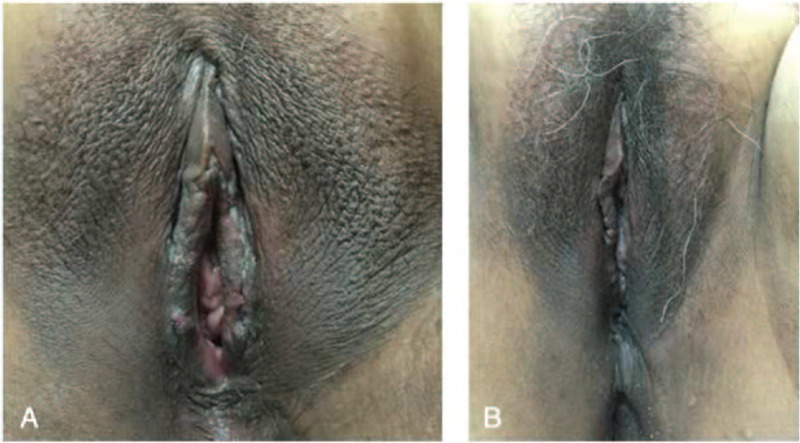
(A) The appearance of postoperative bilateral labia minora (24 hours postoperatively). (B) The appearance of postoperative bilateral labia minora at 18 months postoperatively (we followed up for a total period of 40 months, and the last follow-up was carried out over telephone. The photo shown was taken during an outpatient visit at 18 months postoperatively).

Pathological examination showed that the right-sided lesion was 2.5 × 1.0 cm in size, while the left was 2.5 × 0.8 cm. The surface of the bilateral labia minora lesions was partially covered with proliferative squamous epithelium and displayed papillary hyperplasia. A large number of proliferative sebaceous glands were found under the skin and deep dermis; they were well-differentiated but lacked hair follicles. Immunohistochemical analysis was positive for P63 in the basal layer, vessels positive for cluster of differentiation (CD) 34 and CD31, and negative for S-100 and Sox; 10% of Ki-67 was positive. Thus, the diagnosis was confirmed as NS (Fig. 2A–E). Bilateral labia minora healed well postoperatively (Fig. 3A), and the patient was followed up for 40 months with no recurrence (Fig. 3B).

Written, informed consent was obtained from the patient for the publication of this case report and the attached images.

## 3 Discussion and literature review

NS is common in the scalp and face, and rarely occurs in the trunk, neck, or oral mucosa.^[1]^ In our case, it occurred in the bilateral labia minora, that is far more rare. Because of the location of the lesion, the patient chose to see a gynecologist instead of a dermatologist. The lesions of bilateral labia minora were in the form of papillae with multiple serrated edges, but the tests for human papillomavirus, human immunodeficiency virus, and syphilis were negative, which ruled out sexually transmitted diseases. Considering the rare location and morphological features of the lesion, dermatologists suggested that we removed the lesion, but did not confirm the extent of resection required and the exact preoperative diagnosis. Fortunately, the postoperative pathological report indicated that no residual lesion remained at the resection margin. However, we did not know much about NS before the operation; preoperative biopsy was not performed either. Therefore, the treatment strategy we applied to this patient was flawed. As a remedial measure, we closely followed-up the patient, whose follow-up time has presently reached 40 months since discharge. The patient's bilateral labia minora healed well without recurrence or appearance of other tumors.

For further understanding of NS, we thoroughly searched the available literature using the terms “nevus sebaceous and labia minora” in PubMed, Ovid, China National Knowledge Infrastructure (CNKI), and Web of Science databases and retrieved only 2 related reports.^[6,8]^ To ensure that no reports were missed, we added the terms “(nevus sebaceous) and unusual location” and 6 additional records were found, of which only 1 was ultimately relevant. After reading the above-mentioned articles, we found 2 other related articles,^[9,10]^ though neither had a detailed description of NS. Herein, we list the information we retrieved and sorted (Table 1).

**Table 1:** All cases of nevus sebaceous in genital area.

No.	Reference	Publication time	Country/Region	Department	Gender	Age	Symptom onset	Location	Size	Clinical manifestation	Therapeutic measures	Follow-up time	Outcome
1	Weng C.J^[9]^	1990	Taiwan, China	Department of Plastic Surgery	NM^†^	NM	NM	Genital area	NM	NM	NM	NM	NM
2	Kavak A^[6]^	2008	Turkey	Department of Dermatology/Plastic, Reconstructive and Esthetic Surgery	Female	22	since infancy	Both labia minora	4 × 1 cm	A slight enlargement of the lesion with occasional pruritus	Partial-thickness surgical excision, primary repair	1 year	Neither recurrence nor any tumour development
3	Ugras N^[7]^	2012	Turkey	Pathology Deparment/Obstetrics and Gynecology Deparment	Female	42	2 years ago	Genital localization	1 cm in diameter	A slightly enlarged, sometimes pruritic lesion	Surgery	10 months	No recurrence
4	Hsu M.C^[10]^	2016	Taiwan, China	Department of Dermatology	Female	NM	NM	Pubic area	NM	NM	NM	NM	NM
5	Feito J^[8]^	2018	Spain	Department of Pediatric Surgery	Female	13	9 month ago	Left labia minora	4.4 × 1.5 × 0.8 cm	Hypertrophy and asymmetry of the labia minora pudendi	Surgically removed for treatment	2 days	No further complications
6	This article	2019	China	Department of Gynaecology and Obstetrics	Female	47	2 years ago	Bilateral labia minora	Right: 2.5 × 1.0 cm Left: 2.5 × 0.8 cm	Progressive asymmetrical enlarged bilateral labia minora, slightly tenderness, affecting sexual life and walking	Dermatologist consultation, resection of bilateral labia minora lesion	40 months	Bilateral labia minora healed well without recurrence or other tumor

† NM = not mentioned.

All the cases^[6–10]^ retrieved by us were diagnosed as NS, and they were all located in the genital area. Kavak et al^[6]^ first reported a case of bilateral labia minora NS, for which the patient went to the Department of Dermatology and Plastic, Reconstructive and Esthetic Surgery. The patient sought help due to slight enlargement of the lesion with occasional pruritus. Lesion removal was planned for aesthetic reasons. After excluding the possibility of neurological or ophthalmologic disease and syphilis, the lesion was partially excised and primary repair was achieved. There was no recurrence or development of other tumors at follow-up (postoperative year 1). Ugras et al^[7]^ published the second paper on lesions of NS in the genital region, but the manuscript did not provide accurate details on tumor location. The vulvar biopsy was initially performed in another institution and reported as squamous hyperplasia, whereas a repeat biopsy performed in the author's institution was suggestive of NS. The surgery was performed consequently and the patient was followed up regularly for 10 months without recurrence. Feito et al^[8]^ recently reported a case of NS in 2018. The patient was a 13-year-old girl with hypertrophy and asymmetry of the left labia, but no pain or abnormal sensation. The lesion was surgically removed, and the patient was discharged 2 days later without further follow-up information.

As it is unclear whether the surgical resection was appropriate, we studied the relevant literature further. According to reports,^[11]^ NS is a benign disease with a detection rate of 0.5% to 1% in all dermatologic patients, but it may lead to benign or malignant secondary tumors. Aslam et al^[12]^ noted in a meta-analysis of 4900 NS cases that secondary tumors developed in around 25% of cases. The most common benign tumor was trichoblastoma, while basal cell carcinoma (BCC) was the most common malignant tumor. Hsu et al^[10]^ published a research report on 450 cases, which pointed out that syringocystadenoma papilliferum was the most common benign tumor, accounting for 2.7% of all cases, trichoblastoma occurred in 1.6%, and BCC in about 0.9% of cases. What is worthy of our attention is that this article listed 8 large studies on NS, dated from 2000 to 2016. By analyzing the data reported by Hsu et al, we found that there were 3847 NS cases in total, and 36 cases of BCC accounting for about 0.94%, consistent with other studies. Kamyab-Hesari et al^[13]^ studied the histopathological features of 168 patients with NS in order to determine the proportion of different histological features and incidence of secondary tumors. The results pointed out 9 cases of neoplastic change, including 4 cases of trichoblastoma, 3 of tricholemmoma, and 2 of syringocystadenoma papilliferum. Neither BCC nor other malignant tumors was found. Hence, Kamyab-Hesari holds the view that unnecessary surgical intervention should be avoided, and recommends regular follow-up for children. A prophylactic excision, however, would need thorough evaluation and individualized therapy. Further, Santibanez-Gallerani et al^[14]^ assessed 658 NS pediatric patients under 16 years of age and found no malignancy, but deemed that the risk of NS-induced tumors increased over time. As a supplement, Cribier^[15]^ study of 596 NS subjects recorded 5 cases of BCC, but only in individuals over 22 years of age. Bernard Cribier et al noted that the incidence of BCC may be overestimated because of the similarity between BCC and trichoblastoma, and proposed that when clinical signs indicating malignant transformation were absent, children should not receive preventive surgery. Hsu research results strongly support that malignant transformation of NS mainly occurs in adulthood.^[10]^ When performing prophylactic excision in children, it is an elective procedure and the risks of general anesthesia and surgical complications should be considered. However, resection is strongly advocated during adolescence due to the significantly increased risks. In our opinion, NS is a congenital hyperplastic disease of sebaceous glands, and its treatment should be surgical removal during puberty. The definitive treatment for NS is full-thickness dermal and epidermal excision, and lesions should be excised with 2 to 3 mm margins since it extends at least as deep as the subcutaneous tissue with involvement of adnexal structures.^[16]^ In cases with incomplete clearance, close follow-up is necessary.^[13]^

As for the pathogenesis of NS, Hsu viewpoint deserves our careful consideration. Mosaic mutation of *HRAS* and *NRAS* is responsible for the occurrence of sebaceous nevus, which can activate mitogen-activated protein kinase and phosphoinositide 3-kinase pathways and stimulate cellular proliferation causing so-called “RASopathy” in NS lesional keratinocytes.^[10,12]^ Taking into account that benign and malignant secondary tumors of NS often occur in adulthood, Hsu et al^[10]^ and Idriss et al^[17]^ endorsed the idea that age may be a critical risk factor. Furthermore, Person et al^[18]^ were the first to propose the hypothesis that NS was related to hormonal hyperresponsiveness. They speculated that since sebaceous and apocrine glands are androgen-target organs, androgen receptors may increase in these lesions. To prove this hypothesis, they performed androgen receptor assays in 4 specimens of 3 NS cases, and unsurprisingly, the results showed a significant increase in androgen receptors. NS seems to be directly regulated by hormones, and the level of androgen receptors tends to be up-regulated at all ages,^[10]^ thus being particularly hypersensitive to the effects of hematological androgens.^[19]^

Briefly, NS in genital or other parts is associated with mosaic mutations of *HRAS* and *NRAS* and is accompanied by an increased expression of androgen receptors. According to previous studies, genital NS lesions are usually located on either unilateral or bilateral labia minora, and can present as a slightly pigmented plaque with verrucous lesions or as a yellowish-tan-colored lobulated growth. All cases of NS in the genital area have been reported in female patients so far, with no other skin malignancies and the age of onset ranging from puberty to perimenopausal period concerning treatment strategies, full-thickness skin resection is an effective method, requiring at least 2 to 3 mm margin and close follow-up postoperatively. Whether for aesthetic reasons or for concerns of malignant transformation, with the consent of the patient, we recommend surgical removal of the genital lesion within the period of adolescence or before adulthood. Adolescence may be the best period for surgical intervention owing to a greater risk of malignant change in adulthood. On the other hand, the risk of surgery can be avoided in children, considering the low incidence of malignant transformation in the pediatric age-group. At present, the reported follow-up duration of patients is around 1 year, while we found no recurrence after 40 months of observation. Thus, we strongly believe that if patients with lesion-free margins have no recurrence after 1 year of follow-up, the possibility of recurrence in the future is lower. In addition, differential diagnosis is also very important, by virtue of the fact that patients may visit different departments under different circumstances. Although the diagnosis can be confirmed mainly by pathological examination, detailed history-taking and medical tests should be done to distinguish NS from neurological diseases, syphilis, and other potential diseases.

In conclusion, we recommend full-thickness skin resection of NS lesions with at least 2 to 3 mm margin in the genital area during adolescence or before adulthood, and close follow-up postoperatively. Adolescence may be the best period for surgical intervention owing to a greater risk of malignant change in adulthood. On the other hand, surgical risks can be avoided in children considering the low incidence of malignant transformation in the pediatric age group. In addition, we strongly believe that if patients with lesion-free margins have no recurrence after 1 year of follow-up, the possibility of recurrence in the future is lower. Lastly, we hope that our report can provide some important references to other dermatologists, gynecologists, or plastic surgeons, and remind them to attach enough importance to the long-term follow-up of patients with NS.

## Author contributions

**Conceptualization:** Zhiwei Zhao, Jiang Wu, Yali Miao.

**Data curation:** Qiao Wen, Yanlin Yang, Fengnian Zhao.

**Funding acquisition:** Jiang Wu, Yali Miao.

**Investigation:** Qiao Wen, Ling Wang, Juan Cheng.

**Methodology:** Zhiwei Zhao, Jiang Wu, Yali Miao.

**Project administration:** Zhiwei Zhao, Jiang Wu, Yali Miao.

**Writing – original draft:** Qiao Wen, Zhiwei Zhao.

**Writing – review & editing:** Jiang Wu, Yali Miao.
